# Shared Participatory Research Principles and Methodologies: Perspectives from the USA and Brazil—45 Years after Paulo Freire’s “Pedagogy of the Oppressed”

**DOI:** 10.3390/soc7020006

**Published:** 2017-04-13

**Authors:** Nina Wallerstein, Leandro L. Giatti, Cláudia Maria Bógus, Marco Akerman, Pedro Roberto Jacobi, Renata Ferraz de Toledo, Rosilda Mendes, Sonia Acioli, Margaret Bluehorse-Anderson, Shelley Frazier, Marita Jones

**Affiliations:** 1 Center for Participatory Research, College of Population Health, University of New Mexico, Albuquerque, NM 87131, USA; 2 School of Public Health, University of São Paulo, São Paulo SP 01246-904, Brazil; 3 Institute of Energy and Environment, University of São Paulo, São Paulo SP 05508-010, Brazil; 4 Complexo Educacional Faculdades Metropolitanas Unidas—FMU, Environmental Health Master Degree Program, São Paulo SP 05690-050, Brazil; 5 Federal University of São Paulo, Department of Public Policy and Collective Health, São Paulo SP 11065-240, Brazil; 6 Nursing College, State University of Rio de Janeiro, UERJ, Rio de Janeiro RJ 20.551.030, Brazil; 7 Healthy Native Communities Partnership, Inc., Shiprock, NM 87420, USA

**Keywords:** community-based participatory research, participatory action research, participatory health research, health promotion, empowerment, health inequities, Brazil, United States

## Abstract

The trajectory of participation in health research by community social actors worldwide has been built on a history of community participation from the Ottawa Charter Health Promotion call for community mobilization, to the emancipatory educational philosophy of Paulo Freire, to social movements and organizing for health and social justice. This paper builds on this history to expand our global knowledge about community participation in research through a dialogue between experiences and contexts in two prominent countries in this approach; the United States and Brazil. We first focus on differences in political and scientific contexts, financing, and academic perspectives and then present how, despite these differences, similarities exist in values and collaborative methodologies aimed at engaging community partners in democratizing science and knowledge construction. We present three case studies, one from the U.S. and two from Brazil, which illustrate similar multi-level processes using participatory research tools and Freirian dialogue to contribute to social mobilization, community empowerment, and the transformation of inequitable societal conditions. Despite different processes of evolution, we observed a convergence of participatory health research strategies and values that can transform science in our commitment to reduce health and social inequities and improve community wellbeing.

## 1. Introduction

Since the Ottawa Charter in 1986, community mobilization has been adopted worldwide as a core health promotion strategy, building on multiple World Health Organization documents, including the 1978 Declaration of Alma Ata. Worldwide, these participatory strategies have a longer history in community development and organizing to promote social and economic equity. As part of these efforts, debates abound about the extent of “authentic” participation and whether development should focus on a bottom-up people-centered approach versus top-down economic policies [1,2].

In the research enterprise, community participation has been more recent. Two important and separate historical traditions have been defined: one from the Global South, namely, Latin America, Asia, and Africa, and one from North America and Europe. In this article, we take two prominent countries involved in participatory research from the North and South as exemplars of these traditions. Through dialogue and case studies from Brazil and the United States, we seek to note similarities and differences, unpack discursive narratives, and identify the opportunities for genuine community participation, as well as the potential for co-optation. We will end with recommendations on how to maintain the integrity of community participation in health promotion to promote knowledge democracy, health, and social equity through a research-in-action approach.

Community participation in research has used overlapping but distinct terminology across the globe. Terms have ranged from a focus on action, i.e., action research or action policy research, to a focus on participation, i.e., community-based participatory or collaborative research, or to using both terms as in participatory action or youth participatory action research. Others have focused on participatory mapping methods or on goals, i.e., emancipatory research [3]. While many of these terms are currently used interchangeably, it is important to assess their underlying contexts, values, and intentions to better understand their goals.

Widely understood are two distinct origins and histories; a northern, predominantly U.S., formation and a southern tradition from Latin America, Asia, and Africa [4]. The northern tradition traces its origin to social psychologist Kurt Lewin. In the 1940s–1950s, Lewin identified an action research model of engaging organizational teams in cyclical reflection and action in order to improve institutional practices. The southern tradition emerged in the 1970s from a leftist challenge to ivory-tower academia. Situated in emancipatory “freedom” and participatory language, this model engaged academics with liberation resistance movements and the popular education philosophy of Brazilian educator Paulo Freire.

Today, these different contexts still exist. Within the U.S., the positivist scientific tradition has dominated, making it difficult for community-engaged research (CEnR) and community based participatory research (CBPR) to emerge from the margins until the last few decades. Community participation in research in Brazil, on the other hand, has been more easily recognized as part of the larger adoption of the World Health Organization language of community mobilization and health for all. The approach in Brazil is grounded in a more politicized academia, which seeks to analyze and act on health problems within socio-political contexts. The use of the Portuguese term, “pesquisa-ação” or research-action, also fits well within the Freirian-based social movement of “popular education for health”.

Despite these different country contexts, an emerging perspective of shared principles, dialogical methodologies (often based on Paulo Freire’s writings), and goals to address power differentials has emerged. In the U.S., academic and community partners who have these shared principles and goals remain a small but growing segment of the research enterprise. Internationally, there is also a growing movement towards a unified participatory perspective. With the creation in 2009 of the International Collaborative on Participatory Health Research (icphr.org) and action research networks in other fields, such as the Collaborative Action Research Network (http://carn.org.uk/?from=carnnew/resources.php), researchers from across the globe have sought legitimacy and deeper knowledge about the science and impact of participatory research across multiple sectors, countries, and outcomes in health and education, among other sectors.

In a recent edited volume, Budd Hall and Rajesh Tandon, two of the early founders of participatory research globally, highlight the emerging term knowledge democracy. Knowledge democracy claims the importance of “expertise residing in the world of practice, beyond academia” [5] (p. 26). As an illustration of its growing recognition, the Action Research Network of the Americas is sponsoring the First Global Assembly for Knowledge Democracy in June 2017 in Cartagena, Colombia [6]. It was in Cartagena where Orlando Fals-Borda, one of the original founders of the Southern tradition, hosted the first international symposium on action research forty years previously [7].

This paper will seek to expand our knowledge of community participation in research in public health and health promotion through presenting a dialogue between two major players in this effort; the United States and Brazil. While Brazil and the U.S. are only two examples of these traditions, they provide a rich opportunity to explore debates within the field. We will seek to illuminate each nation’s scientific and political contexts and their specific barriers and facilitators, such as funding and academic demands. We will then discuss the potential for convergence in the emerging international community of CBPR and participatory action researchers who share principles, theories, methods, and intended outcomes of democratized knowledge and social justice. Mini-case studies will add to the dialogue to strengthen thinking on the processes and impacts of participatory research that can be generalized across countries, even as we act within our own specific national contexts.

## 2. United States Context

The U.S. was birthed as a democracy with an ideology of equal opportunity, but in practice its early policies condoned slavery and Native American genocide and have continued to favor the wealthy. As a nation, its early commitment to science was impressive, with the launch of the National Institutes of Health (NIH) at the end of the 19th century. NIH has provided critically-important funding for academic and scientific research yet was also set up to privilege elite university-based research. With a 2016 budget of $32 billion, the NIH funds primarily biomedical laboratory and clinical research. The 1985 Heckler Report, however, from the Secretary of Health and Human Services drew major attention to health disparities in black and minority populations [7]. Since then, in the last decades, there has been growing recognition of the need to reach community populations with health advances, which has led to an upsurge of funding for community engaged research.

A specific social justice-oriented form of community engaged research, called community based participatory research (CBPR), started in the 1990s in public health and health promotion, based on democratic co-creation of knowledge and partnerships [8–10]. This perspective emerged from a history of “maximum feasible participation” federal mandates within the 1960s War on Poverty and from early participatory programs from the Centers for Disease Control and Prevention (CDC). On a community level, it also drew from a long tradition of grassroots place-based labor, civil rights, and other community organizing [11]. CBPR harkens back to the southern tradition, with inspiration from Paulo Freire and Fals Borda, rather than Lewin’s writings [4]. CPBR embraces the philosophy of starting from the strengths of communities and involving them equitably to “combin[e] knowledge and action for social change to improve community health and eliminate health disparities” [12] (p. 4). This perspective was also influenced by many of its practitioners coming out of health promotion, with Dorothy Nyswander’s edict to “start where the people are”. In its first iteration at NIH, environmental “justice” CBPR projects were funded in 1995, spurring other institutes to fund CBPR, including most prominently the Institute for Minority Health and Health Disparities [13]. Freirian empowerment processes and outcomes have continued to be part of much CBPR research in the United States.

Despite CBPR gaining traction among health and health promotion researchers, this approach has been on the margins of the predominantly molecular and clinical research enterprise. With the advent in 2006 of NIH’s major clinical translational science awards (CTSAs) to ~60 Academic Health Centers, community engagement began to enter more of the U.S. research enterprise. One of the major influences behind CTSA adoption was the goal to recruit minorities into research trials to tackle disparities [14]. This more mainstream acceptance has led, most notably, to an increase in “engagement” funding from the Patient Centered Outcomes Research Institute, which mandates involvement of patients, family members, and patient advocates in the research process [15]. A recent CTSA publication of a community engaged research (CEnR) continuum, however, posited an overly broad range of definitions from community outreach through shared leadership. [16]. This inclusion of the uni-directional strategy of outreach, unfortunately, has increased the risk of co-opting the value of equitable involvement of partners.

Thus, in the U.S., the tension continues between a dominant scientific paradigm, which considers the expertise of academics to be most important, versus an egalitarian CBPR paradigm. This debate has been well characterized by Edison Trickett, who posed a dichotomy even within community engagement between a utilitarian versus broad capacity-building worldview model [17]. Trickett suggests that an instrumental approach from academics to fulfilling grant aims not only limits potential impact, but also violates a core CBPR transformation principle of engaging community partners in the democratic creation of knowledge for community benefit and social justice.

The U.S. dominant scientific paradigm is well suited for internal validity studies, which mandate randomized controlled trials as the gold standard for testing the effectiveness of clinical and community interventions. This approach, however, increasingly has been seen as only part of the scientific needed. Implementation scientists have advanced the importance of external validity, with the need to involve community members in designing, adapting, and implementing research appropriately for their diverse populations and settings [18,19]. A growth in team science with mixed methods has also given greater attention to qualitative and hermeneutic understandings of community understandings and strategies for their own well-being. The challenge, however, is how to overcome the dominance of the utilitarian positivist research, which still maintains a research enterprise dominated by academics rather than the co-learning that comes from co-interpretation and community use of data.

Practitioners of CBPR, who espouse power-sharing and partnership, continue to grow in number in the U.S., especially as scholars of color enter the academy and seek to return benefit to their own communities [20]. Communities that have been part of research themselves are also increasingly demanding equal power, which has influenced foundations and government funders to require evidence of collaboration to obtain grants. While NIH grants may still demand utilitarian outcomes, CBPR scholars have found ways to embed social justice agendas in their health promotion research through their long-term commitments and democratic processes of engagement. Research, however, is not immune from the surrounding political context. A new and troubling presidential context in the U.S. has raised grave threats to vulnerable and marginalized groups, including to undocumented immigrants. While too soon to tell, the threatened withdrawal of federal funding in a wide range of areas may portend real threats to CBPR partnerships and health and social equity.

## 3. Brazil Context

Colonial history in Brazil imposed a traditional social structure and economy based on ruling oligarchic families and enterprise. Social participation began to appear in opposition to these structures in the early Twentieth Century. In health, starting in the 1920s, external, mainly U.S., foundations provided a participatory impetus through community-based infectious disease control programs. Later in the 1960s, this foundation funding expanded to other sanitation and educational actions with a participatory model [21,22]. By the 1960s, social participation was at the center of organizing for democracy, which included grassroots movements by workers, students, the landless, indigenous, and other marginalized peoples, as well as organizing for health [23]. At that time, Paulo Freire was using “popular education” to organize cultural literacy circles to democratize citizenship among the urban and rural poor in northeast Brazil under invitation of the president. In 1964, the military coup brutally repressed the democratic fervor of these social movements. Freire was arrested, accused of being a communist, and exiled for 16 years, writing his famous book “Pedagogy of the Oppressed” in exile, with its publication in English in 1970 and in Brazil in 1974 [24–26].

With his exile, Freire became an international figure, espousing the social construction of knowledge and emancipatory learning. His methodology, adopted by many CBPR practitioners in the U.S., Brazil, and beyond, included listening deeply to the needs and issues of the community; creating dialogue for people to critically evaluate and deepen understanding of their own situations within societal conditions of poverty or inequality; promoting actions for people to change those conditions; and then engaging in reflection about their actions in order to start the cycle over again [27]. The constant cycle of reflection, action, reflection enables a commitment to the long-term nature of community participation in the transformation of peoples’ lives.

Post-dictatorship, Brazil’s re-democratization in the 1980’s began a new era for participation. The 1988 federal constitution adopted precepts of social participation and universal access to health, later codified into the democratizing policy of the new Unified Healthcare System (SUS). SUS stimulated both community advisory councils for clinics and social determinants initiatives [23]. Brazilian scientific agencies, such as the National Counsel of Technological and Scientific Development (CNPq) and the São Paulo Research Foundation (FAPESP), following earlier American foundations, began to fund diverse initiatives, spawning Brazilian participatory research currents and critical partnerships [28]. Participatory research, aimed at constructing critical knowledge for political action, was strengthened by Freire’s conception of liberation, in which epistemological and political affinities converged toward reducing oppressive asymmetrical relationships. His popular education pedagogy was founded upon affectivity and people’s knowledge [29].

Some national policy has given an impetus to participation. The 2001 National University Extension Plan [30], calling for knowledge generation based on population needs, gave legitimacy to participatory research for public universities. A National Agenda for Health Research Priorities [31,32], also contained participation within 24 sub-agendas, i.e., in the health of indigenous people, blacks, and women, and in conditions, i.e., mental health, violence, and nutrition among others, but there was no specific agenda for participatory research methodologies [33]. In 2011, the Brazilian National Health Foundation of the Ministry of Health (FUNASA) acknowledged participatory research as a methodology for social mobilization projects specifically focused on environmental health actions and health promotion.

While international standards for health publication favor positivist quantitative (and epidemiologic) research [34], Coimbra [35] argues that much of Latin American academic production in collective health and health promotion is grounded in social-political or social-anthropological orientations. With Latin American discourse is based on applying social-political theory to social democratic practice in the field, he argues that international journal impact rates do not reflect the actual merit of how much Latin American scientific production contributes to policies, intervention strategies, and health programs. Pinto and de Andrade [36] call for extending beyond traditional impact factor metrics of the number of citations to ask what kinds of research have the greater potential to aid humanity. Their concern is that publications in Brazilian journals, with their socio-political orientations, may be seen as lower quality than the majority of biomedical (and positivist-oriented) English-language journals, which would then influence funding in Brazil. In this sense, according to Coimbra:

“Scientific tradition requires time, and a nation such as Brazil, where scientific activity is recent … To give up its scientific independence is to tread the path of imitation; instead of building its own history of development will mean it is doomed to eternal under-development.”. [35] (p. 453)

Akerman [37] extends this argument to call for a genuine “scientific and political will” of researchers to influence policies and therefore to incorporate experiential knowledge and an “experience” metric of how new knowledge has contributed to pathways towards health promotion and healthy public policy development. Research in Brazil too is not separate from its political context. Recent corruption scandals and the impeachment of the democratically-elected president in 2016 have given rise to a worrying context for democracy and equality. Brazilian health promotion academics and practitioners, however, are central and traditional to ongoing social struggles related to equality of women, workers, the landless, and homeless, as well as for maintaining threatened health and social security rights. It is not clear if these struggles will impact research policy “*per se*”, but they do demonstrate a continued history of greater connection between the academic worlds and political movements in Brazil.

Table 1 summarizes the above narration for each country. It offers a side-by-side comparison of the historical and political contexts and government and financial support for participatory research.

## 4. Cases

With this backdrop of political context, government and funding support, and perspectives of participatory research in both countries, we present three mini-case studies to see research-in-action or CBPR in practice. Our objective is not to create a comprehensive view of how participatory research is carried out in each country but to offer illustrative aspects and challenges. We hope to show the importance of the contemporary context as well as the genesis of participation provided by Freirean propositions 45 years ago. Hence, our diverse cases showcase facilitators and challenges related to each country’s context and institutional policies and detail the extent of participatory processes with community members, including their role in the research process itself. We discuss one case study from the U.S., which contains both urban and rural populations, and two case studies from Brazil, one in a remote rural Amazonian setting and one in the center of the largest metropolitan area. We thus offer a pragmatic presentation of differences within each context yet, at the same time, pursue a perspective of common contributions to shared participatory research principles and methodologies.

### 4.1. Healthy Native Community Fellowship (HNCF), United States

Tribal participatory research has been named in the U.S. as a specific form of CBPR that recognizes the sovereignty of tribal nations and their re-assertion of ownership of research on their lands after historic colonization and research exploitation [39]. Tribal communities have faced some of the worst health inequities in the U.S., with high rates of poverty and unemployment, though they also have demonstrable protective factors of culture, social cohesion, and language. In 2005, the Indian Health Service Preventive Task Force established and funded a Native leadership program, using a strengths-based approach with beliefs of community wisdom, cultural knowledge, social justice, and community empowerment to build “Fellow” teams of change agents among American Indian/Alaska Native (AI/AN) tribal nations and urban Native communities [40,41].

The Healthy Native Communities Fellowship (HNCF) has embraced the core health promotion principles from the Ottawa Charter, starting from people’s current conditions and believing in their wisdom to work in multi-disciplinary and multi-sector ways for community wellness. Community mobilization is a core precept, along with strengthening social environments, building personal skills, promoting healthy public policies, and re-orienting services towards prevention and wellness.

Since 2005, fellows have been recruited in multi-sector teams of two to three people (from health, education, senior citizen and environmental programs, police departments, and tribal leadership, among others) to attend a year-long process of three weekly retreats, web-based interactions between retreats [42], and technical assistance to identify key health issues, develop community wellness plans, address policy, and strengthen culture and language in their communities. To date 311 fellows, and 125 teams have been trained by tribes (with populations on average having several thousand members and ranging from a few hundred to the largest Navajo Nation of close to 200,000 members), and by agencies that serve urban Indians. Over 70% of the AI/AN population lives off the reservation in urban centers, though many maintain connections to their tribal lands.

**Figure 1:**
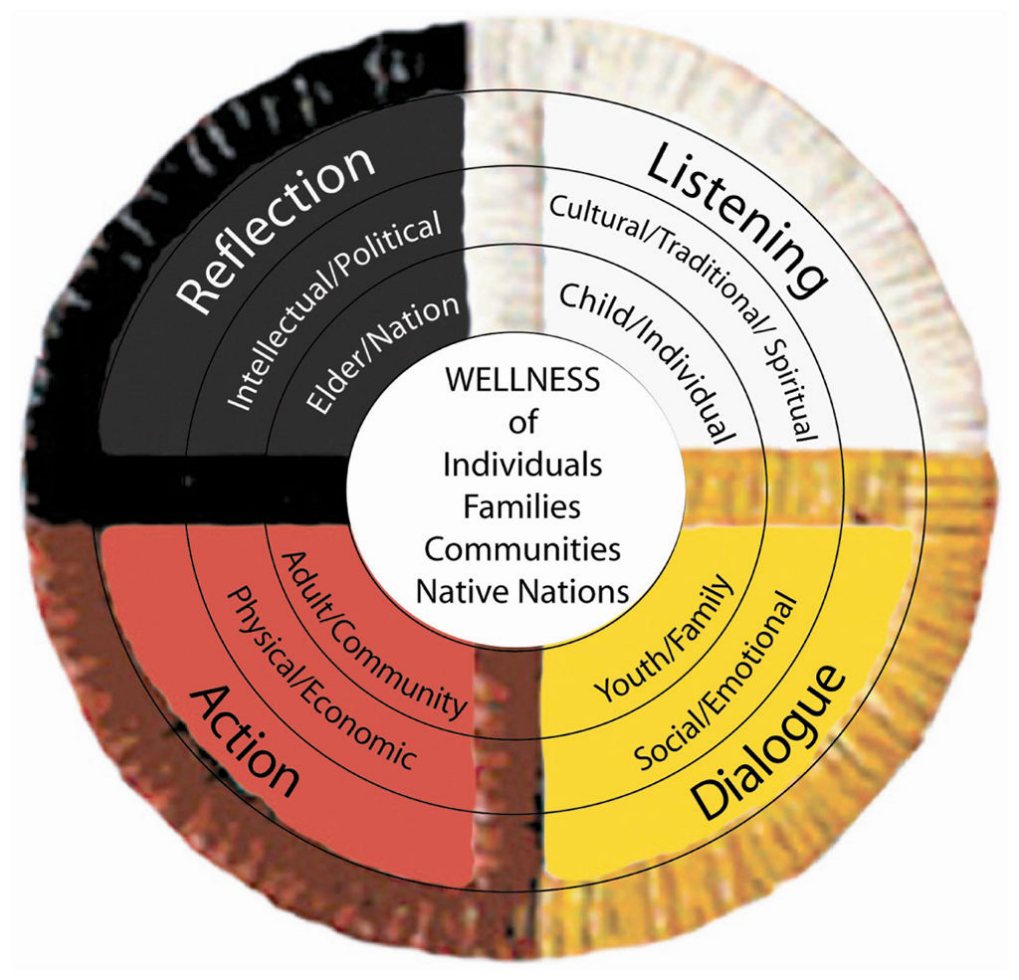
Medicine Wheel.

To embody its principles, curriculum, and leadership skill development, HNCF adopted the four directions Medicine Wheel (see Figure 1 below), which is a spiritual symbol of many U.S. tribal nations, with individual, family, community, and Native nation wellness in the center. The center is surrounded by representations of the four stages of the life cycle, and different concepts of knowledge for each direction. HNCF also adopted the four cyclical stages of the Freirian process of listening/dialogue/action/reflection to facilitate engagement of the Fellows as community researchers of their own issues. In between retreats, fellows use listening surveys, focus groups, and observations to analyze the problems and strengths in their own communities and to develop data for further community dialogues. They bring data and their results of community dialogues back to the Fellowship retreats for assistance in thinking through the next steps of action planning, including working with tribal leadership or other policy-makers for healthy policy change.

As a CBPR research project, HNCF partners with the University of New Mexico Center for Participatory Research (UNM-CPR) and other consultants engaged HNCF staff, fellows, and alumni in developing and refining a quality improvement and participatory evaluation/research process [40,43]. This participatory evaluation/research process has involved HNCF staff in every step of the research process, from defining the core questions, such as how individual fellows are transformed as they engage in changes at a community level, to co-developing qualitative and quantitative instruments, to co-analyzing and interpreting the data for next step program actions. These have included ongoing curriculum revisions for the weekly retreats and, most importantly, seeking to enhance the role of the fellows themselves as researchers of their own community impact.

The CBPR evaluation and theory of change has been multi-level with a three-pronged focus: (1) on individual Fellow transformations (i.e., growth in self-efficacy and empowerment); (2) on teams (cohesion and effectiveness); and (3) on community outcomes (cultural connectedness, wellness events to transform the social environment, and, increasingly, on health-enhancing policies). With ultimate purposes of improving health through social mobilization, this HNCF leadership program epitomizes the transformational purpose of CBPR with a commitment to using participatory methods, Freirian dialogue, and technical assistance from HNCF staff to the communities to create research-in-action that answers the questions and priorities of their communities. Its participatory research methodology was developed outside the positivist clinical trial design and has never followed a randomized control trial design, which historically has posed major challenges in small tribal communities. Indian Health Service funding was flexible in its research design and allowed the partnership to create its own methodology. The participatory approach was essential to integrate western evidence with indigenous cultural and community evidence and knowledge that would be drawn from the communities who were given the responsibility for their own change actions towards improving health and reducing inequities.

Validation of the participatory framework came from following a co-developed theory of change, using mixed methods to triangulate findings, and assuring that the evaluation data was returned to HNCF program staff for their use in improving the delivery of the program and ultimately in testing the effectiveness of the leadership teams in improving wellness in their communities.

As one example, a southwestern tribe in the rural high desert with excess rates of obesity and diabetes has had two teams go through the fellowship training. The “Cultivating Wellness” team has focused on strengthening farming to promote healthy eating and nutrition. Both traditional dry-land methods and spring-irrigated gardens have been tied to family education in healthy cooking and eating. The second team has added a policy dimension to work with their tribal leadership and other non-profits and agencies throughout the region to engage in advocacy against genetically-modified seeds. Their goal is to sustain 16 varieties of heirloom corn as a staple food and foundation of cultural spiritual life. These fellowship teams together have researched the decline of healthy eating in their families through understanding the history of the federal food commodity program, the easy availability of junk foods, and lack of access to commercial healthy fruits and vegetables. They have sought to combat these risks through revitalizing the cultural strengths of traditional agriculture, food knowledge, and language and are working with their local tribal government and social service agencies to engage community members in wellness actions. Teams in urban communities have also addressed obesity and diabetes issues by developing actions on a continuum; from preventive nutrition and physical activity programs, to diabetes care and management, to eliminating “food deserts” in poor neighborhoods by increasing access to healthier foods.

The University of New Mexico Center for Participatory Research continues to work with the non-profit Healthy Native Communities Partnership, which took over the management from Indian Health Service, to capture these empowerment processes and outcomes to support communities to document their successes. Evaluation results have included community change actions, increased collaborations, policy testimonies, changes in individual knowledge and behaviors, programs based on language and cultural strengths, healthier policies, and reduced disparities [41]. The challenges to HNCF are many, including the far-flung distances of fellowship teams across the nation, the variability of local tribal resources to address concerns identified by the fellows, and the inconsistent funding for the fellowship itself. The Healthy Native Community Partnership and the UNM-CPR team, however, recognize the need for ongoing commitment to redress federal policies that have disadvantaged Native communities in the U.S. and the importance of CBPR approaches to build capacity at the local level that hopefully can be long lasting.

### 4.2. Amazonia, Brazil

In Iauaretê, a multi-ethnic indigenous community of 2700 inhabitants undergoing an urbanization process in the Amazon region, an action research approach was undertaken funded by FUNASA. While the funder did not request a participatory methodology within the grant application, University of Sao Paulo (USP) researchers believed that the focus on indigenous health would require an interdisciplinary and participatory approach in order to honor the pluricultural context [44]. The research goal was to support greater social mobilization and community involvement in improving their region’s precarious water supply and lack of sanitation infrastructure.

From 2004 to 2009, there were seven fieldwork campaigns, which overcame many local and shared partnership challenges. These included the long distances (the field was in a remote area of tropical forest located 1200 Km from the state’s capital, Manaus, and more than 4000 Km from São Paulo, where USP is located) and the lack of infrastructure and public investments. Community challenges included the prevalence of mythical elements in health-disease and environmental understanding; the lack of preparedness of the local health care team in health education and health promotion; and the lack of previous social mobilization on water and sanitation issues. Each field work campaign lasted an average of 15 days, spending four days in the round trip. The challenge of creating authentic participation was the first obstacle for the research teams, even though social engagement for the public’s health was a declared constitutional right in Brazil. Sanitation, as a matter of prevention and health promotion, was the focus for the participatory interventions, aimed at raising awareness and conscientization (a Freirian term for the praxis of reflection and action leading to greater understandings and community-based actions). Multiple dialogical participatory tools were used, mainly in the first four field campaigns in 2004 and 2005.

The research-action experience took place through community meetings with researchers working with local indigenous dwellers, using their different fields of knowledge to identify local issues, which enabled constant feedback and adjustment of the research and intervention strategies. The research methodology included the direct participatory tools of engaging indigenous social actors in Freirian dialogue, such as producing “talking maps”, photovoice, and the elaboration of a community newspaper. The talking map consisted of graphic representations of residents’ reality of life, which were collectively produced during community meetings held in each of the 10 villages on two different occasions (constructing current situation and future scenarios) to identify relevant environmental and health characteristics.

Other non-participatory methods were also used, such as surveys of intestinal parasites and analyses of water quality. Although the dialogical methods were clearly more efficient to promote participation, the coupling with non-dialogical tools provided a structure of feedback that was very useful to promote social engagement.

In such a context where there was an enormous gap between popular and scientific knowledge, reflecting asymmetrical relationships among community social actors and researchers, the adaptive iterative feedback created a cyclical process of research and interventions to address the complexity of the environment. Providing education interventions related to the issues that emerged in the research was a parallel primary objective so that local and scientific actors could search together for alternatives to the precarious living conditions. As Santos [45] states, this recognizes the heterogeneous ways of knowing, promoting an ecology of actionable knowledge. In fact, among the indigenous of Iauaretê, Amazonas, there were prevalent and relevant mythological beliefs related to the representations of health, disease, prevention, and cure. Santos’ call for multiple ways of knowledge draws directly on Freirian methodologies of co-constructing social understandings through cyclical processes of dialogue and action. Thus, the concern to reflect multiculturalism in the construction and appropriation of new knowledge was considered indispensable to empower people to address their own issues.

This process contributed to produce contextualized solutions, social mobilization, and empowerment. Despite this success, what should be underscored was the difficulty of engaging governmental managers in this research-intervention, as the remote regional location had both an absence of the state and insufficient social investments to create the necessary water supply and sanitation infrastructure.

According to Flicker [46], participatory interventions can bring tangible and intangible benefits for those involved as well for the community as a whole; otherwise, such benefits can be unequally distributed. Since there were constraints in terms of a public policy and resources for sanitation infrastructure, the engagement of indigenous social actors in the process was the core milestone of the project. The success in this regard was built in the ongoing participatory process, wherein adjustments were continuously undertaken in an adaptive methodology, with outcomes of strengthened social mobilization and empowerment that could reduce the abysmal distance between lay social actors and academics, especially in the context of deep inequalities suffered by indigenous people.

Considering that participatory approaches are always challenging, this experience presented some specific cultural constraints. The population had an initial resistance to the research project, partly due to inertia and a lack of acknowledgement of the water and sanitary risks. While the quantitative assessments of parasites and water quality were critical, the research team found they had to adjust their research tools to recognize cultural knowledge and engage community members in multiple Freirian participatory dialogical processes in order to counter the prevailing welfarism/paternalism. Institutional challenges were the lack of public policies and resources for sanitation infrastructures. In fact, public investment in water and sanitation is completely insufficient, considering the indigenous demands for this basic social right. The processes of social mobilization, however, led to a stronger more symmetrical relationship between community social actors and researchers and brought a common pathway to collaborative knowledge building, local empowerment, and autonomy in terms of a critical stance towards health inequities.

### 4.3. São Paulo, Brasil

Within the city of Sao Paulo (with a population of more than 11 million inhabitants), the sub-municipality of Capela do Socorro is located in the southern region, with a population of 596,000 inhabitants. Capela do Socorro is the largest sub-municipality of São Paulo with a young and poor population, with high rates of violence and homicide. The per capita wage and the level of adult literacy are the lowest compared with the municipality average [47]. The focus of the research was to promote integrated and participatory public management in this low-income context that was suffering from social-environmental injustices. Financed as a policy initiative by the São Paulo Research Foundation (FAPESP), as in the previous example in Brazil, the participatory approach arose from researchers who wanted to forge a partnership between the University of Sao Paulo and the public sub-municipality [48].

In the first phase of research, the “systematization of experience” methodology was adopted [49,50] in order for local public managers and other stakeholders to critically reflect on their experiences and to strengthen their perspectives about how to integrate participatory management with a focus on an improvement of quality of life in the sub-municipality. This method is used to generate new knowledge through integrating empirical and scientific knowledge, objective and subjective dimensions, and theoretical and institutional contexts. The systematization process required a high degree of engagement of the whole group (public managers, researchers, and local inhabitants as a partnership), which created an in-depth review of advances and constraints in public management, notably related to the complex political and administrative decentralization of the large municipality of São Paulo [51].

A second phase, using a leadership mapping method, sought to understand the issues of alliance building and socio-political networks as tools for strengthening social participation. This phase included analyzing the larger social fabric and relationships of power; identification and mapping of groups, movements, organizations and entities operating in the study area, as well as the relationships and networks among them and with the public managers. It was useful to disclose the managerial model complexity using the perspective of socio-political networks as a means to understand how to enhance social participation [52].

Both phases of research were joined with the study of the territory and its problems and strengths to allow an integrated and participatory analysis of management. Thus, it was found that the sustainability of certain innovative and participatory management approaches, involving intersectoral practices and social collaboration, cannot be effective unless they are combined with decentralized administrative measures able to ensure autonomy of the sub-municipality at the local level. Challenges were also identified within the sub-municipality, such as a lack of resources and poor qualifications and knowledge among the different levels of social actors. These led to much dialogue and education by the USP faculty about the purpose of the investigation and the opportunity therefore to openly discuss the challenges and ambiguities for local public managers in assuming a participatory management stance. Considering the large metropolitan dimensions of São Paulo, the recognition of sub-municipality autonomy has been vital for public managers to engage in the contradictory processes of participatory management. Their side-by-side dialogues with community actors has led to an evolving process of achieving common understandings for defining priorities and attaining actions on these priorities [53].

## 5. Discussion and Conclusions

Despite very different contexts between the United States and Brazil in terms of their histories and funding for participatory research, investigators in both countries have shown a coming together of principles, social justice motivations, and use of Freirian dialogical research methodologies.

### 5.1. Context, Funding, and Academic Agendas: Brazil

The two examples from Brazil illustrate some contemporary features of carrying out participatory research in this country, including the facilitators and challenges in the interactions between researchers, funders, managers, and local social actors. While the majority of funding sources still do not cover participatory processes, there remains a strong influence of Ottawa Charter concepts, including community mobilization, within health promotion research. Participatory research methodologies are more likely to be accepted with educational interventions in more isolated minority groups, which suffer social exclusion and vulnerability, or with those that focus on social justice. With greater understanding of the complexity of social-environmental and health problems, Brazilian funding agencies increasingly are supporting interdisciplinary research and therefore the involvement of community social actors who can bring their local knowledge to the table. In the case of working with indigenous communities through FUNASA, their funding stream did recognize the importance of financing a participatory research methodology.

With both Brazilian projects, the researchers wrote participatory research proposals and engaged in Freirian participatory dialogue methods to co-construct knowledge, as they perceived the added value of the knowledge gained in collaboration. The Brazilian cases also illustrated the political imperative and politicized stance of academic researchers to understand inequalities as subverting democracy. Researchers in each case therefore promoted the empowerment and democratic involvement of social actors against oppressive forces and conditions. They sought new knowledge that could translate into actions, which could target institutional policies and practices that perpetuated inequalities. Though their outcomes fell short of policy change, the equitable involvement of community social actors led to greater collective understanding about social conditions and was a step in the direction of knowledge democracy.

### 5.2. Context, Funding and Academic Agendas: United States

The U.S. CBPR project, which crosses both urban and rural geographies, engages fellows and fellow teams in Freirian participatory dialogical methods of listening/dialogue/action and reflection processes of research. With partnership support between the University of New Mexico and the HNCF staff, local teams identify health priorities in their native communities, use mixed methods to research information based on their priorities, and develop health promotion action strategies for community and policy change. Through a deeply-embedded cultural process, the teams are able to integrate academic evaluation methodologies with indigenous knowledge. Although different teams have had different levels of success over the years in transforming policies or practices around health inequities, the skills and cultural embeddedness of the HNCF trainings have promoted greater recognition of the wisdom and knowledge that comes from communities. This wisdom has been used to strengthen the capacity of teams to take on health promotion initiatives over the long haul. While HNCF has not been funded by the National Institutes of Health, its multi-method research approach is similar to other CBPR NIH projects that are based in robust theoretical designs with goals to address health disparities. In general, NIH-funded projects do not assume a politicized academic stance, and U.S. public health research is often less-theoretically grounded than that from Brazil. The longer term existence and higher levels of U.S. NIH funding, however, as opposed to Brazil, has offered multiple avenues for community engaged research and CBPR projects.

The challenge in the U.S. remains in the academic view that community engagement is less rigorous scientifically than more controlled methodologic designs that can prove the effectiveness of evidence-based interventions. Implementation science has challenged this perspective with new pragmatic trial designs, among others, although NIH grants still privilege randomized control trials. Despite this tension, a strength of U.S. science has been the recognition of the importance of tight methodologic reasoning, and community engaged research has profited from this expectation. Community engaged research has gained in prominence with meta-analyses and systematic reviews that show effectiveness in health behavior changes, as well as changes in community capacities, more culturally-relevant interventions, and greater community ownership over the long haul to tackle inequities.

### 5.3. Commonalities between Brazil and the U.S

While there are differences in research and academic context as well as in financial support in each country, the two communities of participatory health researchers (investigators using the terms research-in-action in Brazil and CBPR in the U.S.) have many more dimensions in common than might be expected. Within the U.S., it is the smaller network of CBPR researchers within a larger community engagement continuum which has staked out their commitment to social justice, knowledge democracy, and the reduction of health inequities as their driving force. While NIH projects may not be looking for social justice justification, CBPR researchers have become skilled at writing strong methodologic grants targeting health outcomes yet still embracing the larger goals of building capacity to address social determinants.

Both sets of researchers from each country recognize the importance of deep and broad community involvement throughout the research process, whether as a core principle from the Ottawa Charter (in Brazil) or from the classic CBPR principles, as written by Barbara Israel and colleagues in 1998 (in the U.S.) [54]. Both embrace a social constructivist view of the importance of co-constructing reality with community partners contributing their community and cultural knowledge as equal to academic knowledge. Methodologically, this means an openness to the integration of qualitative and quantitative inquiry, reflexivity of partners to their own processes, and the creation of research designs that embrace diversity of knowledge development. Participatory and reflexive processes can guide projects in terms of interactions among academic researchers, professionals/public managers, and community members/local social actors in order to produce symmetric and collaborative knowledge, apply new knowledge to address social determinants, and create conditions that enable racial minorities and other disenfranchised communities to become empowered.

In both countries, there is an ability to learn from and support Freirian “educação and conhecimento popular” (or popular education and knowledge) such as understanding indigenous and other community members’ cultural and experiential knowledge as contributing to the research process. This is a significant re-claiming of the key philosophical base of Paulo Freire’s “Pedagogy of the Oppressed”. Freire wrote about conscientization (or conscientização) as the processes of working with people for them to uncover their own knowledge, to construct their own social understanding of reality, and to create their own strategies for change. This deep recognition of and respect for the knowledge of the people makes possible the symmetric interaction among lay social actors and educators (for Freire) or, in the case of researchers, among lay social actors with academic researchers. This collaborative interaction based on respect can challenge traditional structures of oppression that support deep inequities and can strengthen health promotion’s contribution to knowledge democracy as a true social justice enterprise. The Brazilian cases more directly emphasize the goal of joining research knowledge with social movements, although this is a still challenge since the publication of Freire’s “Pedagogy of the Oppressed”.

In the U.S., a research effort of over ten years has had multiple stages to further the science of academic-community research collaborations, starting in 2006 with the development of a CBPR conceptual model [55]. A mixed methods NIH study of 200 federally funded partnerships (2009–2013) identified measures of practices and outcomes from the model [56] and uncovered promising practices and associations of these practices with a range of research, community transformation, and health equity outcomes [57,58]. This study then has been followed by the NIH-funded Engage for Equity Study (2015–2020) [59]. Engage for Equity has refined the measures, conducted new internet surveys (also translated into Spanish) of an equal number of U.S.-based partnerships, and is developing new tools and resources to support partnerships to engage actively in self-evaluation. While seeking to add to the scientific literature, Engage for Equity also has sought to maintain its value base of democratizing knowledge and social justice. Its basic hypothesis is that “reflexivity matters”; that partnerships benefit by assessing their strengths and challenges of working together and by seeking promising practices to improve their effectiveness. Engaging in reflexive dialogue can empower all participants to recognize that contributing their knowledge is necessary to create societal change. The Engage for Equity study has also sought to assess key outcomes, including shared power in research, increased cultural centeredness of interventions within communities, and healthy policy changes, as well as more long-term health outcomes. The CBPR model on which the studies are based has been translated into Spanish and Portuguese and is being adopted in multiple global settings.

In conclusion, the experiences and discussion of the historical process of participatory research in the USA and in Brazil can provide important perspectives in global understanding of participatory health research and health promotion research. Though only two countries, these shared participatory research principles and methodologies in the explored contexts offer concrete examples for others to consider from the North and the South. These examples may especially help others who wish to adopt Freirian dialogical methodologies for the transformation of oppression and inequities, forces that still threaten human health in vulnerable contexts. As a global force of participatory researchers, enhancing the value of participatory health research processes within the mainstream scientific research agenda is critically important for reaching our goals to improve health and social equity.

## Figures and Tables

**Table 1:** Determinants of community participation in research U.S.A./Brazil.

USA Historical and Political Context	Brazil Historical and Political Context
Democratic origins (DeToqueville) Historical myth of equality vs. reality of favoring the wealthy and corporations Individualism predominant, though strong history of community organizing Late 1800s, NIH launched with focus on laboratory and clinical sciences, with randomized controlled trials seen as gold standard Positivist quantitative research privileged Political discourse more in margins within academia 1980s: health disparities appear in government documents. More attention to implementation, community engagement, team science and qualitative approaches Current decade: Highest rates of health inequities and poverty which provides basis for continued CBPR research to combat inequities	Oligarchic origins Early 20th Century fighting for democracy as fragile Pre and post-dictatorship: History of social movements of landless/workers/peasants/students (ideologies include: liberation theology, Marxism, popular education) Reality of Dictatorship: 1964–1985 Post Dictatorship: 1990: Lei 8142: Law of Participation of Social Control, i.e., Councils for SUS health posts [38]; Political and social justice discourse high within academia Evaluation of “participation” entered research agenda in health promotion Epidemiology and other quantitative empirical science understood as more valued by international journals Academic struggles to highlight values of qualitative and participatory approaches
**USA Government and Foundation Support**	**Brazil Government and Foundation Support**
Dept of Health and Human Services Heckler Report on Minority and Black Health, in 1985, opened space for CBPR as research to address disparities [12]. Early 1960s CDC funding for participatory health processes First National Institute of Environmental Health Science funding in 1995 for environmental justice CBPR grants; then extended to other NIH Institutes Clinical Translational Science Awards (CTSA) in 2006 opened space to incorporate community engagement CTSA continuum with outreach as engagement strategy runs risk of maintaining academic control vs. egalitarian CBPR model Community demands provoke sponsor and funder responses Social justice agendas often hidden within NIH grants	Starting in 1920s with international foundations funding initiatives to control infection diseases, then in 1960s bringing social participation side by side with wellbeing. Post dictatorship, in late 1980’s a new democratic perspective associated with public policy and federal constitution principles. >2000: Financing of government for evaluating participation; Investigators transformed research approaches to also be participatory Research funding at much lower scale than U.S.
